# Real-life data of Pasireotide LAR in acromegaly: a long-term follow-up

**DOI:** 10.1007/s40618-023-02275-1

**Published:** 2024-01-20

**Authors:** C. Urbani, F. Dassie, B. Zampetti, R. Mioni, P. Maffei, R. Cozzi, F. Bogazzi

**Affiliations:** 1 Endocrinology Unit, Department of Medicine, Azienda Ospedaliero-Universitaria Pisana, Pisa, Italy; 2 Department of Medicine, University of Padua, Padua, Italy; 3 Endocrine Unit, Grande Ospedale Metropolitano Niguarda-Milano, Milan, Italy; 4 Department of Clinical and Experimental Medicine, University of Pisa, Pisa, Italy

**Keywords:** IGF-1, Headache, PAS-LAR, GH

## Abstract

**Objective:**

Pasireotide LAR (PAS-LAR) was released in Italy in 2017 to treat acromegaly patients resistant to SRLs (Somatostatin Receptors Ligands). The long-term follow-up data of PAS-LAR therapy in Italy are limited. This study aimed to evaluate the efficacy and safety of PAS-LAR in acromegaly.

**Design:**

Patients with acromegaly in PAS-LAR treatment were enrolled in three tertiary Italian endocrinological centers and evaluated by a retrospective observational real-life multicentre study.

**Methods:**

Patients have been studied before (baseline) and 1, 6, 12, 24 and > 36 months after PAS-LAR start. Clinical, biochemical, and pituitary magnetic resonance data were collected, along with information on adverse events. Acromegaly disease activity was classified according to the IGF-1 index (normal value < 1.0).

**Results:**

Fifty patients (female 23) were enrolled. PAS-LAR treatment (mean follow-up 24 ± 16 months) significantly decreased IGF-1 levels (IGF-1 index baseline vs last visit: 1.9 ± 0.6 vs 1.2 ± 0.6, *p* < 0.0001). At the last visit, 67% of patients had controlled disease, and 44% showed a decrease in tumor volume. Clinical and biochemical efficacy was observed as early as after 1-month of PAS-LAR treatment (IGF-1 index baseline vs 1-month: 1.9 ± 0.6 vs 1.4 ± 0.7, *p* < 0.0001). Also, 50% of patients referred headache improvement or disappearance. Fifteen patients discontinued PAS-LAR due to failure of treatment and poor glycaemic control. The prevalence of diabetes increased from 33% at the baseline to 54% at the last visit (*p* = 0.0072).

**Conclusion:**

In real-life settings, PAS-LAR significantly decreases symptoms, IGF-1 levels, and the size of adenoma in patients with acromegaly resistant to SRLs. Beneficial effects may occur early after the first injection.

**Supplementary Information:**

The online version contains supplementary material available at 10.1007/s40618-023-02275-1.

## Introduction

Pasireotide LAR (PAS-LAR) is a multireceptor-targeted somatostatin analogue that is recommended as a second-line medical treatment for acromegaly. PAS-LAR is characterized by a higher affinity for somatostatin receptor type 5 and type 2 and due to its binding profile, it has been shown to have a higher clinical efficacy in reducing GH excess compared to first-generation somatostatin analogues (SRLs) [1–3].

Data from clinical trials showed that the biochemical response to PAS-LAR was significantly greater compared to that of octreotide LAR (OCT-LAR) in patients naive to medical treatment. Furthermore, both PAS-LAR and OCT-LAR induced a significant reduction in pituitary tumour mass [4, 5]. Two studies also confirmed the clinical and radiological efficacy, the double-blind PAOLA trial (C2402) and its extension study, which compared PAS-LAR with maximal SRLs treatment dosage [6–10]. Regarding safety issues, clinical trials showed mild to moderate hyperglycaemia within the first 3 months of treatment, and this adverse event (AE) rarely led to the discontinuation of PAS-LAR treatment [6, 7, 11]. Lastly, the efficacy and safety of PAS-LAR combined treatment with Pegvisomant (PEGV) were also evaluated by the PAPE study, which showed a significant reduction in PEGV dose in patients under combined treatment with the possibility of a definitive discontinuation of PEGV during the extension phase [12, 13].

In the literature, only a few papers studied efficacy and safety in real-life scenarios. Although these studies have confirmed PAS-LAR treatment efficacy with good biochemical response in 20–54% of patients, however, in contrast to previous clinical trial data, real-life studies showed a significant deterioration of glucose metabolism that may require an early and aggressive treatment. Only one recent—long-term real-life study—also showed a potential delayed effect on control of GH and IGF-1 excess by PAS-LAR [14–18].

Real-life studies are crucial for tailoring acromegaly treatment and identifying patients who are most likely to benefit from PAS-LAR treatment [19–21]. PAS-LAR was licensed in Italy in 2017 and real-world data on its efficacy and safety are currently lacking. The primary endpoints of this study were to assess the biochemical and radiological efficacy of PAS-LAR treatment and to establish its long-term safety in a real-life clinical setting across three tertiary care Italian endocrinological centers.

## Material and methods

### Study design and patient population

This retrospective multicentre observational study was conducted by the Endocrinology section of the Department of Clinical and experimental Medicine of the University of Pisa (PI), the Division of Endocrinology of ASST Grande Ospedale Metropolitano Niguarda in Milan (MI), and the Internal Medicine Unit 3 of the Department of the Medicine of Padua University Hospital (PD).

Fifty consecutive patients treated with PAS-LAR between 2017 and 2020 were enrolled. Inclusion criteria were patients under PAS-LAR treatment (40 mg starting dose of PAS-LAR via intramuscular injection every 28 days) in both monotherapy and combined therapy, older than 18 years, regardless of the previous type of acromegaly treatments.

The primary aims of the study were to determine the long-term efficacy and safety of PAS-LAR in the real life and to assess the time course of response to PAS-LAR. Secondary outcomes of the study were to evaluate the effects of PAS-LAR on the dimension of pituitary GH-secreting adenoma and in the symptoms of acromegaly as well as to describe the factors that led to PAS-LAR and discontinuation in real practice.

Enrolled patients have been evaluated before PAS-LAR start during previous treatments (baseline) and 1, 6, 12, 24, 36, and > 36 months after PAS-LAR start. For each patient, the following data have been collected: age of acromegaly diagnosis, GH nadir during oral glucose tolerance test (OGTT) at diagnosis, type and number of neurosurgery approach, type and timing of radiotherapy treatments, acromegaly medical treatments before the start of PAS-LAR, any ongoing acromegaly medical treatment combined with PAS-LAR therapy, other medical treatments, in particular type 2 diabetes mellitus treatments, and indications for PAS-LAR treatment.

The dose of PAS-LAR or reasons for PAS-LAR discontinuation have been collected at each time point of evaluation. Dose changes or PAS-LAR discontinuation decision was at the discretion of the treating endocrinologist. Symptoms of acromegaly (headache, perspiration, paraesthesia, osteoathralgia, and fatigue) were also collected, using a Likert scale spanning between 0 and 5 points.

For every patient, the following biochemical data have also been collected: GH mean of three point at diagnosis, at baseline and at each evaluation timepoint except for those patients under PEGV treatment, IGF-1 values, and IGF-1 index (we calculated the IGF-1 index by dividing the IGF-1 value by the age- and sex-specific upper normal limit) at diagnosis, at baseline, and at each evaluation timepoint [22]. Serum levels of GH and IGF-1 were measured in the morning in fasting conditions, using different assays. The authors decided to classify the activity of the acromegaly disease according to the IGF-1 index: IGF-1 index less than 1.0 for controlled disease and greater than 1.0 for active disease. We also recorded data on glucose metabolism, such as fasting glucose (mmol/L) and HbA1c (mmol/mol) at every timepoint evaluation.

Pituitary magnetic resonance (MRI) data have also been collected: adenoma size (microadenoma, macroadenoma, empty sell) at diagnosis, at baseline, and when available MRI data were reported at the corresponding time point of evaluation during PAS-LAR treatment. The timing of imaging follow-up was at the discretion of the treating endocrinologist. Adenoma dimension changes (reduction or increase volumes) were reported when described in the neuroradiologist report.

Drug-related adverse events have been registered and classified according to the Common Terminology Criteria for Adverse Events (CTCAE).

This study was conducted in accordance with the Declaration of Helsinki. Written informed consent was obtained from all acromegaly patients. The authors adhered to good clinical practice guidelines. The study was approved by the Padua Local Ethics Committee (number 5520/AO/22).

### Statistical analyses

We used the G*Power platform (version 3.1.9.4) and the SPSS statistical package, version 25 (IBM Software Group) to determine sample size and perform data analysis.

The sample size for our study was determined using the G*Power platform (version 3.1.9.4). We based the sample size on the observed rate of biochemical control of acromegaly in the PAOLA study for subjects treated with PAS-LAR (both 40 and 60 mg/28d), which was 18.46%, compared to the same subjects during SRLs treatment where the rate was 0.00%. We used an alpha value of 0.15 and a sample power of 0.85 for this calculation. Based on these parameters, the minimum required sample size was 50 participants.

We assessed the normality of continuous data and the homogeneity of variance using the Shapiro–Wilk and Levene tests, respectively. For normally distributed continuous data, we reported mean ± standard deviation (SD). Non-parametric data and ordinal variables were presented as the median and interquartile range (IQR). Dichotomous and qualitative values were expressed as numbers and percentages. Differences between the three Institutions were assessed by ANOVA test, Kruskal–Wallis test and χ2 test for homogeneity, as appropriate. Significant or nearly significant variables were explored post-hoc through Tukey’s, Dunn’s, and Fisher’s tests, as appropriate.

To compare disease control features at baseline and during different time points of PAS-LAR treatment we used mixed-effect analysis and the Kruskal–Wallis test for continuous and categorical variables, respectively. Post hoc analyses for multiple comparisons have been performed using the Tukey’s multiple comparisons test or the the two-stage linear step-up procedure of Benjamini, Krieger and Yekutieli, as appropriate. The same approach has been used to compare glycaemic features of enrolled subjects.

Differences in clinical symptoms scores between baseline and the last visit were evaluated using the Wilcoxon rank sum test. A two-sided *p* value of < 0,05 was considered statistically significant.

## Results

Table 1 presents the baseline and clinical characteristics of the 50 acromegaly patients at diagnosis and at the start of PAS-LAR treatment. At diagnosis, 92% of the patients had a macroadenoma, with a mean GH of 17.7 ± 21.8 ug/L after OGTT and an IGF-1 index of 3.4 ± 1.4. All patients had previously received treatment with SRLs, with 16 patients treated with high-dose SRL (OCT-LAR 40 mg every 4 weeks in 2 patients, Lanreotide -LAN- 120 mg every 3 weeks in 4 patients, and LAN 120 mg every 2 weeks in 10 patients. Notably all patients were resistant to previous SRLs treatment, and 86% had received multimodal treatments for acromegaly (Table 1). The mean time from acromegaly diagnosis to PAS-LAR start was 8 years in these patients. The mean IGF-1 index at baseline was 1.9 ± 0.6, 12% of the patients had an IGF-1 ULN ≤ 1.3. One patient had a biochemical controlled disease and the reason for PAS-LAR start was related to severe headache resistant to SRL. The mean follow-up time was 24 ± 16 months. Details on statistical analysis of baseline and clinical characteristics are shown in Tables 1SM, 2SM, and 3SM.

**Table 1:** Demographic and clinical features at the enrolment (overall and single center populations)

	Overall [*n* = 50]	PI [*n* = 18]	PD [*n* = 17]	MI [*n* = 15]	*p* value
Female gender,	23 (46)	8 (44)	9 (53)	6 (40)	0.754^†^
Age at Diagnosis, *yr*	43 ± 11 [50]	48 ± 11 [18]	40 ± 8 [17]	41 ± 12 [15]	0.050^‡^
*Diagnosis of acromegaly*					
Pulsatile GH, *ng/mL*	21.0 {10.0–36.8} [47]	15.0 {9.1–29.4} [18]	17.6 {6.0–35.5} [14]	34.0 {15.0–54.0} [15]	0.079^§^
GH nadir during OGTT, *ng/mL*	17.7 ± 21.8 [30]	13.8 ± 8.5 [16]	13.4 ± 12.9 [11]	54.0 ± 57.7 [3]	0.006^‡^
IGF-1-index	3.4 ± 1.4 [45]	3.2 ± 0.8 [18]	2.7 ± 0.9 [12]	4.1 ± 1.8 [15]	0.016^‡^
Time to diagnosis*, yr*	7 {5–10} [46]	8 {6–9} [18]	4 {2–10} [13]	7 {3–10} [15]	0.720^§^
Macroadenoma*,*	46 (92)	18 (100)	16 (94)	12 (80)	0.100^†^
*Adenoma secretion class*					
Pure GH,	47 (94)	17 (94)	17 (100)	13 (87)	0.159^†^
Mixed GH/PRL,	1 (2)	1 (6)	0 (0)	0 (0)	
Mixed GH/TSH,	2 (4)	0 (0)	0 (0)	2 (13)	
*Previous treatments*					
Surgery,	35 (70)	11 (61)	15 (88)	9 (60)	0.130^†^
First Generation SST analogues,	50 (100)	18 (100)	17 (100)	15 (100)	–
Dopamine agonists,	7 (14)	2 (11)	5 (29)	0 (0)	0.052^†^
GH receptor antagonist,	23 (46)	8 (44)	5 (29)	10 (67)	0.106^†^
Radiotherapy,	7 (14)	2 (11)	3 (18)	2 (13)	0.853^†^
At least two treatments,	43 (86)	15(83)	16 (94)	12 (80)	0.476^†^
*Acromegaly activity at enrolment*					
Random GH, *ng/mL*	2.8 {1.2–5.7} [39]	5.3 {1.9–8.0} [11]	1.3 {0.8–2.7} [13]	3.9 {1.2–5.7} [15]	0.057^§^
IGF-1-index	1.9 {1.5–2.3} [50]	1.9 {1.7–2.4} [18]	1.4 {1.3–1.6} [17]	2.2 {2.0–2.7} [15]	< 0.001^§^

Quantitative continuous data are expressed as mean ± SD. If data do not follow a normal distribution, then the median and interquartile range are reported. Dichotomous and categoric data are shown as numbers and (percentages). For quantitative variables, the sample size is detailed by square brackets. *P*-values were used to explore the differences observed between the three enrolment centers (PI, PD, MI) for each variable. Analyses have been conducted by the χ2 test for homogeneity (^†^), one-way ANOVA test (^‡^), and Kruskal–Wallis test (^§^), as appropriate. Pegvisomant treated patients at the enrolment have been excluded from random GH analysis

### PAS-LAR treatment: doses and titration

In the majority of cases, patients started PAS-LAR therapy due to persistent disease activity despite receiving the maximum dosage of SRLs (88% of the patients). Other reasons for starting PAS-LAR included pituitary adenoma progression (10% of the patients), PEGV intolerance (4% of the patients), and clinically poorly controlled headaches in 4 patients (8%). The mean time between diagnosis and the start of PAS-LAR treatment was 10 ± 8 years. The initial dose of PAS-LAR was 40 mg every 28 days in all patients.

The three centers followed a similar approach to PAS-LAR dose titration. The dose of PAS-LAR was increased to 60 mg every 28 days in cases where IGF-1 levels were significantly above the normal limit for age and sex. Conversely, the dose was decreased to 20 mg every 28 days when IGF-1 levels fell below the lower limit of normal. During the study period, the PAS-LAR dose was escalated to 60 mg every 28 days in 17 (34%) patients and was decreased to 20 mg every 28 days in 7 (14%) patients.

PAS-LAR was combined with cabergoline in two patients (in one patient cabergoline was present at PAS-LAR start due to a mixed GH-PRL adenoma) and with PEGV in four patients. In one case, the dose of PEGV was reduced after PAS-LAR start.

**Fig. 1:**
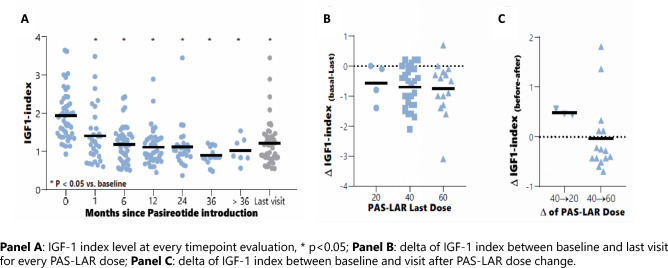
Effect of PAS-LAR on IGF-1 index

During the study, 18/50 (36%) patients discontinued PAS-LAR due to lack of disease control in 14/18 cases (78%), severe hyperglycaemia in 12/18 cases (67%), hypoglycaemia in 1 patient and 2 patients for other reasons (see Supplemental Material Table 4SM). Detailed PAS-LAR dose titration is shown in Fig. 1SM and Table 5SM of the supplemental material.

### PAS-LAR efficacy

The effect of PAS-LAR on the biochemical control of acromegaly and delta of PAS-LAR dose titration are depicted in Fig. 1 and summarized in Table 2. PAS-LAR treatment led to a significant decrease in IGF-1-index levels as early as 1 month after the start of the treatment (baseline vs 1-month: 1.9 ± 0.6 vs 1.4 ± 0.7, *p* = 0.001). At the last visit, the IGF-1 index was significantly decreased compared to baseline (baseline vs the last visit: 1.9 ± 0.6 vs 1.2 ± 0.6, *p* < 0.0001). Fifty-three percent of the patients achieved an IGF-1 index ≤ 1.3, and 35% demonstrated disease control (IGF-1 index ≤ 1.0) at the 1-month visit. At the last visit, 70% of the patients had an IGF-1 index less than 1.3 (*p* < 0.0001 vs baseline), and 42% had an IGF-1 index of less than 1.0 (*p* < 0.0001 vs baseline). The IGF-1-index and the percentage of patients with disease control or IGF-1 normalization did not differ significantly between the time points considered. The delta IGF-1-index between the baseline and 1-month visit and between baseline and the last visit were  – 0.6 ± 0.7 and  – 0.8 ± 0.8, respectively. The delta IGF-1 index of the 1-month visit vs the last visit was  – 0.2 ± 0.8. Only minor changes in the IGF-1 index were observed by continuing the treatment and escalating the dose, without achieving statistical significance (Fig. 1; Fig. 2SM Table 4SM). Baseline GH levels significantly decreased at the last visit compared to baseline (GH: 4.3 ± 5.2 vs 1.7 ± 2.1 *p* < 0.05). Fifty-two percent of the patients had a GH level of less than 1.0 ug/L at the last visit.

**Table 2:** Disease control during PAS-LAR treatment

	Overall [*n* = 50]	PI [*n* = 18]	PD [*n* = 17]	MI [*n* = 15]	*p* value
*IGF-1-index*					
Baseline	1.9 ± 0.6 [50]	2.0 ± 0.5 [18]	1.6 ± 0.6 [17]	2.3 ± 0.6 [15]	0.003^‡^
1 month	1.4 ± 0.7 [34]^γ^	1.5 ± 0.7 [14]	1.4 ± 0.8 [8]	1.3 ± 0.8 [12]	0.887^‡^
6 months	1.2 ± 0.5 [49]^δ, D^	1.2 ± 0.5 [17]	1.2 ± 0.5 [17]	1.2 ± 0.5 [15]	0.856^‡^
12 months	1.1 ± 0.5 [43]^δ, D^	1.2 ± 0.6 [17]	1.1 ± 0.4 [15]	1.0 ± 0.4 [11]	0.779^‡^
24 months	1.1 ± 0.5 [33]^δ, E^	1.0 ± 0.4 [14]	1.3 ± 0.7 [13]	1.1 ± 0.4 [6]	0.271^‡^
36 months	1.0 ± 0.3 [19]^δ, F^	0.9 ± 0.3 [10]	1.0 ± 0.2 [7]	0.8 ± 0.0 [2]	0.626^‡^
> 36 months	0.9 ± 0.3 [12]^γ, F^	0.8 ± 0.3 [8]	1.4 ± 0.2 [3]	0.8 [1]	0.026^‡^
Last visit of the patient	1.2 ± 0.6 [50]^δ^	1.1 ± 0.6 [18]	1.3 ± 0.6 [17]	1.2 ± 0.5 [15]	0.746^‡^
*Random GH*					
Baseline	4.3 ± 5.2 [38]	5.1 ± 3.4 [11]	2.2 ± 2.3 [13]	5.7 ± 7.5 [14]	0.199^‡^
1 month	2.1 ± 1.7 [27]^D^	2.5 ± 2.0 [6]	2.4 ± 2.5 [6]	1.9 ± 1.2 [15]	0.693^‡^
6 months	2.1 ± 2.2 [39]^D^	1.9 ± 1.1 [8]	2.1 ± 2.9 [16]	2.1 ± 1.8 [15]	0.960^‡^
12 months	1.8 ± 2.0 [31]^D^	2.0 ± 2.5 [10]	1.9 ± 2.2 [10]	1.5 ± 1.3 [11]	0.834^‡^
24 months	1.6 ± 1.9 [24]^β, D^	1.6 ± 2.2 [10]	1.7 ± 1.8 [9]	1.4 ± 1.7 [5]	0.952^‡^
36 months	0.8 ± 0.5 [16]^β, F^	0.9 ± 0.4 [10]	0.9 ± 0.7 [4]	0.4 ± 0.2 [2]	0.320^‡^
> 36 months	0.8 ± 0.4 [10]^F^	0.8 ± 0.3 [8]	1.3 [1]	0.5 [1]	–
Last visit of the patient	1.7 ± 2.1 [42]^α^	2.0 ± 2.6 [15]	1.5 ± 1.6 [12]	1.7 ± 1.9 [15]	0.824^‡^
*IGF-1-index ≤ 1.3*^§^					
Baseline, *n*° (%)	6 (12)	2 (11)	4 (24)	0 (0)	0.123^†^
1 month, *n*° (%)	18 (53)^γ, C, D, E^	6 (43)	4 (50)	8 (67)	0.471^†^
6 months, *n*° (%)	32 (65)^δ, D^	10 (59)	11 (65)	11 (73)	0.689^†^
12 months, *n*° (%)	31 (72)^δ^	11 (65)	11 (73)	9 (82)	0.610^†^
24 months, *n*° (%)	26 (79)^δ^	12 (86)	10 (77)	4 (67)	0.620^†^
36 months, *n*° (%)	18 (95)^δ^	9 (90)	7 (100)	2 (100)	0.622^†^
> 36 months, *n*° (%)	11 (92)^δ^	8 (100)	2 (67)	1 (100)	0.195^†^
Last visit of the patient	35 (70)^δ^	14 (78)	12 (71)	9 (60)	0.539^†^
*IGF-1-index ≤ 1.0*^§^					
Baseline, *n*° (%)	1 (2)	0 (0)	1 (6)	0 (0)	0.371^†^
1 month, *n*° (%)	12 (35)^β, D^	4 (29)	3 (38)	5 (42)	0.776^†^
6 months, *n*° (%)	18 (37)^γ,^	5 (29)	7 (41)	6 (40)	0.739^†^
12 months, *n*° (%)	21 (49)^δ^	7 (41)	9 (60)	5 (46)	0.549^†^
24 months, *n*° (%)	15 (46)^δ^	6 (43)	6 (46)	3 (50)	0.956^†^
36 months, *n*° (%)	11 (61)^δ^	7 (70)	2 (33)	2 (100)	0.169^†^
> 36 months, *n*° (%)	7 (58)^γ^	6 (75)	0 (0)	1 (100)	0.054^†^
Last visit of the patient	21 (42)^δ^	7 (39)	6 (35)	8 (53)	0.555^†^
*Random GH ≤ 1,0*^§^					
Baseline, *n*° (%)	9 (24)	1 (9)	5 (39)	3 (21)	0.234^†^
1 month, *n*° (%)	11 (41)	2 (33)	3 (50)	6 (40)	0.838^†^
6 months, *n*° (%)	16 (41)	3 (38)	8 (50)	5 (33)	0.625^†^
12 months, *n*° (%)	16 (52)^α^	5 (50)	6 (60)	5 (46)	0.795^†^
24 months, *n*° (%)	13 (54)^β^	6 (60)	3 (33)	4 (80)	0.217^†^
36 months, *n*° (%)	10 (63)^β^	6 (60)	2 (50)	2 (100)	0.474^†^
> 36 months, *n*° (%)	7 (70)^β^	6 (75)	0 (0)	1 (100)	0.240^†^
Last visit of the patient	22 (52)^β^	9 (60)	5 (42)	8 (53)	0.635^†^
*IGF-1-index ≤ 1,0 and random GH ≤ 1,0*^§^					
Baseline, *n*° (%)	0 (0)	0 (0)	0 (0)	0 (0)	–
1 month, *n*° (%)	6 (25)^α, D, E^	1 (17)	2 (33)	3 (25)	0.801^†^
6 months, *n*° (%)	9 (23)^α, D, E^	1 (13)	4 (25)	2 (27)	0.724^†^
12 months, *n*° (%)	11 (36)^γ^	3 (30)	5 (50)	3 (27)	0.503^†^
24 months, *n*° (%)	7 (29)^β^	3 (30)	2 (22)	2 (40)	0.780^†^
36 months, *n*° (%)	8 (50)^γ^	5 (50)	1 (25)	2 (100)	0.233^†^
> 36 months, *n*° (%)	6 (60)^δ^	5 (63)	0 (0)	1 (100)	0.335^†^
Last visit of the patient	13 (31)^α^	5 (33)	2 (17)	6 (40)	0.415^†^

IGF-1, IGF1-index and GH values were analyzed using mixed-effects analysis for multiple comparisons, with a fixed effect for the time of visit (IGF-1 index *p* < 0.0001; GH *p* = 0.0023). Kruskal–Wallis test was employed to assess differences between visits in terms of rate of acromegaly control, measured by the normalization of IGF-1 index (IGF-1 < 1,0, *p* < 0,0001; IGF-1 < 1,3, 0,0001) values, random GH levels (*p* = 0,0272), or both (*p* = 0,0002). Greek characters (α, β, γ, δ) indicate significance compared to the baseline (α: *p* < 0.05; β: *p* < 0.01; γ: *p* < 0.001; δ: *p* < 0.0001). Latin characters (A, B, C, D, E, F) indicate statistical significance between different visits (A: vs 6-month visit; B: vs 12-month visit; C: vs 24-month visit; D: vs 36-month visit; E: vs > 36-month visit; F: vs last visit). ^§^Percentages were calculated based on the number of subjects with available IGF-1 index and/or GH values for the specific visit. *p* values were used to explore the differences observed between the three enrolment centers (PI, PD, MI) for each variable. Analyses have been conducted by the χ2 test for homogeneity (^†^) or one-way ANOVA test (^‡^), as appropriate

PAS-LAR efficacy analyses were also performed excluding patients that started combined treatment with PEGV or cabergoline during the study. At the last visit, the IGF-1 index was significantly decreased compared to baseline (baseline vs the last visit: 1.9 ± 0.6 vs 1.2 ± 0.6, *p* < 0.001). At the last visit, 66% of the patients had an IGF-1 index less than 1.3 (*p* < 0.0001 vs baseline), and 44% had an IGF-1 index of less than 1.0 (*p* < 0.0001 vs baseline).

An escape phenomenon (patient with IGF-1 index > 1.3 and treatment with PAS-LAR 60 mg) were found in 2 patients but one of them had only a transient disease control of 6 months.

Seven patients underwent radiotherapy treatment (three patients with gamma-knife, three patients with cyberknife, one patient with conventional treatment). The mean time between radiotherapy treatment and PAS-LAR study enrollment was 8.57 ± 10.59 years. The radiotherapy did not impact on disease control during PAS-LAR treatment (data analysis can be found in Table 8SM of supplemental material).

Significant improvements were observed in key symptoms of acromegaly during PAS-LAR treatment, particularly for headache, fatigue, arthralgia and perspiration (baseline vs last visit score: 66 vs 12 *p* = 0.04, 79 vs 39 *p* = 0.002, 87 vs 54 *p* = 0.042, 47 vs 17 *p* = 0.02, respectively). However, there was no significant improvement in paraesthesia (baseline vs last visit score: 31 vs 10 *p* = NS). Among patients who experienced headaches at baseline (*n* = 22), 64% reported rapid improvement or disappearance of headaches even within a few days after the first dose of PAS-LAR (based on the first-month visit after the baseline report). Further details regarding clinical symptoms of acromegaly during follow-up can be found in Tables 6SM, 7SM, and Fig. 3SM of the supplemental material.

Pituitary MRI scans were available for a subset of 37 out of 50 patients (74% of our cohort). Among these patients, 43% demonstrated a decrease in tumour volume, while 54% had a stable pituitary adenoma (Table 9SM supplemental material).

### PAS-LAR Safety

Hyperglycaemia was the most commonly observed AE associated with PAS-LAR treatment. Fasting glucose and HbA1c levels showed a significant increase from baseline to the last visit (baseline vs last visit: fasting glucose 104 ± 21 vs 126 ± 32 mg/dL *p* < 0.0001; Hba1c 40 ± 6 vs 47 ± 8 mmol/mol, *p* < 0.0001). The prevalence of diabetes also increased from 38% at baseline to 56% at the last visit (*p* = 0.0072). Forty-six percent of patients required the introduction or intensification of hyperglycaemia treatments. The following classes of antidiabetic drugs were used to start or intensify hyperglycaemia treatment: biguanides in 17 patients, GLP-1 receptor agonist in 3 patients, DPP-4 inhibitors in 7 patients, SGLT2 inhibitors in 1 patient, rapid acting insulin in 4 patients, and long-acting insulin in 4 patients. It is worth noting that glucose metabolism deteriorated early following the introduction of PAS-LAR, with no statistically significant differences in glucose parameters observed between the 1-month visit and the last visit during the follow-up period. Two patients developed diabetic ketoacidosis and twenty-three patients required the initiation or intensification of DM treatment. The prevalence of diabetes, fasting glucose and HBA1c levels, and Type 2 DM treatments are shown in Table 3, Tables 7SM, 10SM, 11SM, 12SM, 13SM, and Fig. 4SM in the supplementary material. Analysis on glucose metabolism excluding patients in combined treatments showed a significant increase of fasting glucose and HbA1c from baseline to the last visit (baseline vs last visit: fasting glucose 104 ± 21 vs 126 ± 31 mg/dL *p* < 0.0001; HbA1c 40 ± 6 vs 47 ± 8 mmol/mol, *p* < 0.0001). The prevalence of diabetes also increased from 38% at baseline to 50% at the last visit (*p* < 0.001). Short-term (6 months) PAS-LAR dose adjustments (up-titration and down-titration) significantly affected glycemic and HbA1c levels (Table 10SM).

**Table 3:** Glucose abnormalities at baseline and during the follow-up in the overall population [*n* = 50]

	Baseline	1 month	6 months	12 months	24 months	36 months	< 36 months	Last visit
Fasting glucose mg/dl [*n*]	104 ± 21 [50]	123 ± 30 [33]^γ†^	126 ± 28 [43]^δ^†	123 ± 25 [37]^δ^	118 ± 26 [30]^αF^	126 ± 26 [18]^α^	128 ± 25 [12]	126 ± 32 [44]^†δ^
Hba1c mmol/mol [*n*]	40 ± 6 [50]	45 ± 10 [34]^γ^	45 ± 9 [47]^δ†^	46 ± 7 [41]^δ^	44 ± 7 [31]^αF^	46 ± 8 [19]^β^	48 ± 8 [12]^α^	47 ± 8 [48]^δ†^
Glucose abnormalities [*N*]	[50]	[37]	[49]	[43]	[33]	[19]	[12]	[50]
Normal glucose tolerance, *n*° (%)	19 (38)	6 (16)	7 (14)	7 (16)	5 (15)	1 (5)	1 (8)	7 (14)
Prediabetes conditions, *n*° (%)	20 (40)	14 (38)	20 (41)	16 (37)	11 (33)	9 (47)	4 (33)	15 (28)
Diabetes mellitus, *n*° (%)	11 (22)	17 (46)	22 (45)	20 (47)	17 (51)	9 (47)	7 (58)	28 (56)

Fasting glucose and HbA1c were analyzed using mixed-effects analysis for multiple comparisons, with a fixed effect for the time of visit (Fasting glucose *p* < 0.0001; HbA1c *p* =  < 0.0001). Kruskal–Wallis test was employed to assess differences between visits in terms of glycaemic status (nomal, pre-DM, and DM, *p* value of the test 0,0051). ^†^Patient excluded with severe hyperglycaemia (two patients with fasting glucose over 500 mg/dL and one patient with HbA1c over 100 mmol/mol). Greek characters are used to indicate the significance vs baseline (α *p* < 0.05; β *p* < 0.01; γ *p* < 0.001; δ *p* < 0.0001). Latin characters indicate a statistical significance among the different visits (A vs the 6 months visit; B vs the 12 months visit; C vs the 24 months visit; D vs 36-months visit; E vs the > 36 months visit, F vs the last visit)

The second most commonly reported AE was mild gastrointestinal discomfort. No cases of symptomatic cholelithiasis were observed. Five patients (10%) reported mild nausea (grade 1/2), ten patients (21%) experienced mild to moderate diarrhoea (grade 1/2), and nineteen patients (56%) had moderate to severe diarrhoea (grade 3/4). Other reported AEs include sinus bradycardia, alopecia and insomnia all observed in only one case. Interestingly, most adverse events were recognized in the first-month visit or following a dose escalation.

## Discussion

This real-life study aimed to evaluate the efficacy and safety of PAS-LAR in acromegaly patients who were unresponsive to previous treatments. PAS-LAR demonstrated significant reductions in IGF-1 levels and pituitary adenoma size. Efficacy of PAS-LAR treatment was clearly seen at one month evaluation, suggesting a very fast response on GH and IGF-1 hypersecretion, which persisted throughout the follow-up period. Notably, PAS-LAR therapy effectively improved acromegaly symptoms, particularly headache. At the first evaluation, after 1 month of PAS-LAR treatment, acromegaly patients referred a sudden improvement of this symptom even in several cases of serious and disabling headache. The most commonly reported adverse events associated with PAS-LAR treatment were hyperglycemia and mild gastrointestinal side effects. These adverse events, including metabolic disturbances, were observed early, often within the first-month of treatment [1, 2].

We documented an early significant decrease in the IGF-1 index within the first-month of treatment. Sixty-six percent of the patients had a controlled disease after 6 months of  PAS-LAR therapy and 39% had an IGF-1 index less than 1.0 combined with a GH level lower than 1.0 ug/L. Therefore, the rate control of acromegaly was higher than  what was described in clinical trials and other real-life clinical studies [17]. As reported in other studies, acromegaly disease control was also maintained in the long-term follow-up [18]. In some patients, we observed a delayed response that was not associated to the PAS-LAR escalation dose and this finding, already described in other studies, was probably influenced by a more responsiveness of somatostatin receptors over the years. The increased response to PAS-LAR treatment could lead to a dose reduction during long-term follow-up [18, 23]. In our cohort, the radiotherapy treatment that the patients performed before and during the observation period did not influenced the disease control, probably due to the long follow-up between radiotherapy treatment and PAS-LAR start. Six patients were on combined treatment with cabergoline or PEGV by the end of the follow-up period. In our cohort, differently from PAPE studies, combined treatment with PAS-LAR and PEGV was not associated with a reduction in PEGV dose, potentially due to the selection of patients with a more severe disease in our cohort [12, 13].

PAS-LAR also proved effective in symptom control, with significant improvements observed in fatigue and sweating from the basal evaluation to the last follow-up visit. Notably, PAS-LAR exhibited early control of intractable headaches, which are often debilitating in acromegaly patients (20). In our cohort, PAS-LAR produced early control of headache that persisted even in the long-term follow-up. This rapid response suggests that the analgesic effect of PAS-LAR may be mediated by somatostatin analogue receptors sst1, sst2, sst4, and sst5, as demonstrated in the animal models [24]. Notably, headache in our patients was not responsive to previous treatment with SRLs or other medical treatments, suggesting that the specific PAS-LAR sst profile may mediate the analgesic and anti-inflammatory effects. Some authors suggested that the truncated somatostatin receptor 5 may modulate therapy and headache response in patients with acromegaly [25]. Since the analgesic effect appeared very early—within the first-month of treatment—we suggest that it was not primarily related to a significant pituitary adenoma shrinkage.

The most frequently reported AE was hyperglycemia, followed by gastrointestinal symptoms such as diarrhoea and nausea. Other isolated events included sinus bradycardia, alopecia, and insomnia. Hyperglycemia was an early AE, and in some cases, it led to the prevalence of pre-diabetes and diabetes being higher than that reported in other cohorts. Hyperglycaemia was an early AE and at the last visits the prevalence of pre-diabetes and diabetes was higher than those described in the long-term Israeli cohort [14]. Appropriate management with diabetes medications helped to control hyperglycemia, but in 10 patients, PAS-LAR was discontinued due to this AE. These findings emphasize the need for careful glucose control from the start of PAS-LAR treatment. Despite the risk of hyperglycemia, the study suggests that PAS-LAR should be considered in cases of severe, PAS-LAR-responsive headaches or biochemical normalization of acromegaly activity.

The main limitations of this study are the retrospective design, the multicenter design with the use of different assays for the measurement of IGF-1, GH, glucose and HbA1c, and the MRI scheduled according to good clinical practice in the three centers. Moreover, the biochemical control of acromegaly may also be influenced in the long term by radiotherapy or combined treatments.

In conclusion, this real word study conducted across three tertiary care centers confirms that PAS-LAR therapy is an effective treatment option for acromegaly patients resistant to other medical therapies as well as for those patients with uncontrolled headache. Biochemical efficacy on disease control and adverse events were observed early even during the first month after the start of PAS-LAR, and they remained stable also in the long-term follow-up. These data suggest that the first-month evaluation should be considered part of the standard of care of naïve PAS-LAR acromegaly patients.

### Supplementary Information

Below is the link to the electronic supplementary material.

Supplementary file1 (DOCX 885 KB)

## Data Availability

All data set is available on request. The data of this study are guarded anonymously by Dr. C. Urbani of Pisa University Hospital.
