# Supplementary material for: Real-life data of Pasireotide LAR in acromegaly: a long-term follow-up
Source: J Endocrinol Invest. 2024 Jan 20;47(7):1733–41. doi: 10.1007/s40618-023-02275-1 (PMC11196287; doi:10.1007/s40618-023-02275-1)

**SUPPLEMENTAL MATERIAL**

**TAB1SM Multiple comparisons for qualitative and dichotomous variables of Table 1 & 1SM.**

|  | **χ2 test for homogeneity**  **p-value** | **PI vs PD**  **p-value** | **PI vs MI**  **p-value** | **PD vs MI**  **p-value** |
| --- | --- | --- | --- | --- |
| **Female gender,** | 0.754 |  |  |  |
| **Diagnosis of acromegaly** | | | | |
| Macroadenoma | 0.100 | 0.486 ‡ | 0.083 ‡ | 0.319 ‡ |
| **Adenoma secretion class** | 0.159 |  |  |  |
| **Previous treatments** | | | | |
| Surgery, | 0.130 |  |  |  |
| First Generation SST analogues, | - |  |  |  |
| Dopamine agonists, | 0.052 | 0.228 ‡ | 0.489 ‡ | 0.046 ‡ |
| GH receptor antagonist, | 0.106 |  |  |  |
| Radiotherapy, | 0.853 |  |  |  |
| At least two treatments, | 0.476 |  |  |  |
| **Cause for PAS-LAR start** | | | | |
| Uncontrolled acromegaly, | 0.021 | 0.019 ‡ | 0.455 ‡ | 0.178 ‡ |
| Morphological progression, | 0.083 | 0.338 ‡ | 0.108 ‡ | 1.000 ‡ |
| Poor controlled headache, | 0.015 | 0.045 ‡ | - | 0.104 ‡ |
| Other treatments side effects, | 0.553 |  |  |  |
| **Months of study completed** | | | | |
| 1 month | - |  |  |  |
| 6 months | 0.404 |  |  |  |
| 12 months | 0.209 |  |  |  |
| 24 months | 0.040 | 0.927 ‡ | 0.038 † | 0.070 † |
| 36 months | 0.043 | 0.505 † | 0.027 † | 0.080 ‡ |
| > 36 months | 0.031 | 0.146 † | 0.015 ‡ | 0.349 ‡ |
| **PAS-LAR dose at Last visit** | 0.330 |  |  |  |
| **Patients discontinuated PAS-LAR** | 0.405 |  |  |  |

Significant or close to significant variables (p≤0,1) at the χ2 test for homogeneity have been submitted to multiple comparison analyses using the χ2 test (†) or Fisher’s exact test (‡), as appropriate

**TAB2SM Multiple comparison test for quantitative continuous variables of Table 1 & 1SM.**

|  | **ANOVA summary** | | **Tukey’s *post-hoc* multiple comparisons tests** | | | | | |
| --- | --- | --- | --- | --- | --- | --- | --- | --- |
|  | **F-value (df)** | **p-value** | **PI vs PD** | | **PI vs MI** | | **PD vs MI** | |
|  |  |  | **Mean Diff. (95% CI)** | **Adj. p-value** | **Mean Diff, (95% CI)** | **Adj. p-value** | **Mean Diff, (95% CI)** | **Adj. p-value** |
| **Age at Diagnosis, *yr.*** | 3,201 (2,47) | 0,050 | 8,3 (-0,2 to 16,8) | 0,055 | 6,8 (2,0 to 15,6) | 0,155 | -1,5 (-10,4 to 7,4) | 0,910 |
| **Diagnosis of acromegaly** | | | | | | | | |
| GH nadir during OGTT, ng/mL | 6,298 (2,27) | 0,006 | 0,4 (-17,7 to 18,5) | 0,999 | -40,2 (-69,3 to -11,0) | 0,006 | -40,5 (-70,7 to -10,4) | 0,007 |
| IGF1-index | 4,577 (2,42) | 0,016 | 0,5 (-0,6 to 1,7) | 0,450 | -0,9 (-2,0 to 0,2) | 0,112 | -1,5 (-2,6 to -0,3) | 0,014 |

Df (between groups, within groups)

**TAB3SM Multiple Comparison tests for continuous data of Table 1 & 1SM not following a normal distribution.**

|  | **Kruskal-Wallis test** | **Dunn's multiple comparisons test** | | | | | |
| --- | --- | --- | --- | --- | --- | --- | --- |
|  | **p-value** | **PI vs PD** | | **PI vs MI** | | **PD vs MI** | |
|  |  | **Mean Rank diff** | **Adjusted p-value** | **Mean Rank diff** | **Adjusted p-value** | **Mean Rank diff** | **Adjusted p-value** |
| **Diagnosis of acromegaly** | | | | | | | |
| Pulsatile GH, ng/mL | 0,079 | -0,8 | 1,000 | -10,0 | 0,112 | -9,2 | 0,212 |
| Time to diagnosis, yr. | 0,720 |  |  |  |  |  |  |
| **Time from diagnosis to enrolment, yr.** | 0,041 | -12,1 | 0,043 | -8,7 | 0,266 | 3,4 | 1,000 |
| **Acromegaly activity at enrolment** | | | | | | | |
| Random GH, ng/mL | 0,057 | 10,4 | 0,078 | 2,3 | 1,000 | -8,1 | 0,185 |
| IGF1-index | <0,001 | 13,5 | 0,018 | -6,3 | 0,641 | -19,9 | <0,001 |
| **Duration of treatment at last visit** | 0,007 | 6,6 | 0,548 | 16,1 | 0,005 | 9,5 | 0,193 |
| **Time to discontinuation of PAS-LAR, mos.** | 0,297 |  |  |  |  |  |  |

**TAB4SM. Delta IGF-1 index in disease control during PASLAR treatment.**

|  | **Overall [n=50]** | **PI [n=18]** | **PD [n=17]** | **MI [n=15]** | **p-value** |
| --- | --- | --- | --- | --- | --- |
| **Δ IGF1-index between the last and baseline visit** | -0.8±0.8 [50] | -0.9±0.7 [18] | -0.3±0.4 [17] | -1.1±1,0 [15] | 0.005 ‡ |
| **Δ IGF1-index between the last and 1-month visit** | -0.2±0.8 [34] | -0.4±0.7 [14] | 0.0±0.5 [8] | -0,1±1,0 [12] | 0.525 ‡ |
| **Δ IGF1-index between the last and first* visit** | -0.1±0.7 [50] | -0.4±0.6 [18] | 0.1±0.5 [17] | -0,1±0,9 [15] | 0.149 ‡ |
| **Δ IGF1-index between the 1-month and baseline visit** | -0.6±0.7 [34] | -0.6±0.7 [14] | -0.2±0.6 [8] | -1,0±0,6 [12] | 0.046 ‡ |
| **Δ IGF1-index between the first* and baseline visit** | -0.7±0.6 [50] | -0.6±0.6 [18] | -0.4±0.5 [17] | -1,1±0,6 [15] | 0.007 ‡ |
| **Variation of IG1-index according to PAS-LAR dose (baseline-Last visit)** | | | | | |
| 20 mg / 28d | -0.7±0.8 (4) |  |  |  |  |
| 40 mg / 28d | -0.8±0.7 (28) |  |  |  |  |
| 60 mg / 28d | -0.7±0.8 (18) |  |  |  |  |

The delta (Δ) of the IGF-1 index was calculated as the difference between the IGF-1 index observed at two specific visits of interest. For instance, to compute the Δ IGF-1 index between the last visit and the baseline visit, follow these steps: Subtract the IGF-1 index at the baseline visit (subtrahend) from the IGF-1 index at the last visit (minuend). Consequently, positive Δ values signify an increase in the variable between the two time point, while negative values denote a decrease. * The first visit refers to the initial visit following the introduction of PAS-LAR. For 34 patients, it corresponds to the 1-month visit, while for 16 subjects, it corresponds to the 6-month visit. P-values were used to explore the differences observed between the three enrolment centres for each variable. Analyses have been conducted by the χ2 test for homogeneity (†) or one-way ANOVA test (‡), as appropriate.

**Tab5SM. Summary of treatment duration, dose adjustments and details on PAS-LAR discontinuations**

|  | **Overall [n=50]** | **PI [n=18]** | **PD [n=17]** | **MI [n=15]** | **p-value** |
| --- | --- | --- | --- | --- | --- |
| **Months of study completed** | | | | | |
| 1 month | 50 (100) | 18 (100) | 17 (100) | 15 (100) | - |
| 6 months | 49 (98) | 17 (94) | 17 (100) | 15 (100) | 0.404 † |
| 12 months | 43 (86) | 17 (94) | 15 (88) | 11 (73) | 0.209 † |
| 24 months | 33 (66) | 14 (78) | 13 (77) | 6 (40) | 0.040 † |
| 36 months | 19 (38) | 10 (56) | 7 (41) | 2 (13) | 0.043 † |
| > 36 months | 12 (24) | 8 (44) | 3 (18) | 1 (7) | 0.031 † |
| **Duration of treatment at last visit, *mos.*** | 24 {12-37} [50] | 35 {19-50} [18] | 25 {17-34} [17] | 12 {6-24} [15] | 0.007 § |
| **PAS-LAR dose adjustments, *n.*** | | | | | |
| Dose reduced from 40 to 20 mg/28d. | 7 | 2 | 4 | 1 | - |
| Dose increased from 40 to 60 mg/28d. | 17 | 7 | 5 | 5 | - |
| Dose reduced from 60 to 40 mg/28d. | 3 | - | 2 | 1 | - |
| Dose increased from 20 to 40 mg/28d | 2 | 1 | 1 | - |  |
| **PAS-LAR dose at Last visit** | | | | | |
| 20 mg/28d. | 4 (8) | 1 (5) | 3 (18) | 0 (0) | 0.330 † |
| 40 mg/28d. | 28 (56) | 10 (56) | 10 (59) | 8 (53) |  |
| 60 mg/28d. | 18 (36) | 7 (39) | 4 (24) | 7 (47) |  |
| **Patients discontinued PAS-LAR** | 18 (36) | 8 (44) | 4 (24) | 6 (40) | 0.405 † |
| **Time to discontinuation of PAS-LAR, mos.** | 12 {6-24} [36] | 18 {12-26} [8] | 13 {5-25} [4] | 6 {6-14} [6] | 0.297 § |
| **Causes for stopping of PAS-LAR** | | | | | |
| Lack of disease control | 14 (78) | 6 (75) | 3 (75) | 5 (83) | - |
| Severe hyperglycaemia | 12 (67) | 5 (63) | 3 (75) | 4 (67) | - |
| Hypoglycaemia | 1 (6) | 1 (13) | 0 | 0 | - |
| Pituitary Surgery | 2 (11) | 2 (25) | 0 | 0 | - |
| Others | 2 (11) | 1 (13) | 0 | 1 (17) | - |

Quantitative continuous data are expressed as mean ±SD. If data do not follow a normal distribution, then the median and interquartile range are reported. Dichotomous and categoric data are shown as numbers and (percentages). For quantitative variables, the sample size is detailed by square brackets. P-values were used to explore the differences observed between the three enrolment centres for each variable. Analyses have been conducted by the χ2 test for homogeneity (†), one-way ANOVA test (‡), and Kruskal-Wallis’s test (§), as appropriate. Multiple comparisons among the centres for statistically significant and nearly significant variables are detailed in Supplementary Tables 3SM to 5SM. For the cause of PAS-LAR stop we considered the number of patients that have discontinued the treatment as a denominator for percentages.

**TAB6SM. Symptom’s questionnaire**

|  | **Baseline [n=44]** | **Last visit [n=44]** | **p-value** |
| --- | --- | --- | --- |
|  | **Mean (IQ range)** | **Mean (IQ range)** |  |
| **Headaches** | 1 (0 to 3) | 0 (0 to 2) | 0.007 |
| **Perspiration** | 0 (0 to 2) | 0 (0 to 0) | 0.003 |
| **Paraesthesia** | 0 (0 to 1) | 0 (0 to 1) | 0.400 |
| **Arthralgia** | 2 (1 to 3) | 1 (0 to 3) | 0.042 |
| **Fatigue** | 2 (0 to 4) | 0 (0 to 2) | <0.001 |
| Wilcoxon signed-rank test | | | |

**TAB7SM Kruskal Wallis test for Assessing Qualitative Parameters of Acromegaly's Biochemical Control and Glucose Metabolism Status. Multiple Comparisons**

|  | **IGF1-index ≤1,3**  **p-value** | **IGF1-index ≤1,0**  **p-value** | **Random GH ≤1,0**  **p-value** | **IGF1-index & random GH ≤1,0**  **p-value** | **Glucose abnormalities**  **p-value** |
| --- | --- | --- | --- | --- | --- |
| **Baseline vs 1-month visit** | 0,0003 *** | 0,0041 ** | 0,1285 | 0,0260 * | 0,0063 ** |
| **Baseline vs 6-months visit** | <0,0001 **** | 0,0002 *** | 0,0873 | 0,0179 * | 0,0030 ** |
| **Baseline vs 12-months visit** | <0,0001 **** | <0,0001 **** | 0,0125 * | 0,0006 *** | 0,0039 ** |
| **Baseline vs 24-months visit** | <0,0001 **** | <0,0001 **** | 0,0120 * | 0,0094 ** | 0,0021 ** |
| **Baseline vs 36-months visit** | <0,0001 **** | <0,0001 **** | 0,0058 ** | 0,0001 *** | 0,0069 ** |
| **Baseline vs >36-months visit** | <0,0001 **** | 0,0003 *** | 0,0063 ** | 0,0001 *** | 0,0072 ** |
| **Baseline vs Last visit** | <0,0001 **** | <0,0001 **** | 0,0055 ** | 0,0012 ** | 0,0001 *** |
| **1-months visit vs 6-months visit** | 0,2865 | 0,6172 | 0,9818 | 0,8664 | 0,9857 |
| **1-months visit vs 12-months visit** | 0,0693 | 0,1659 | 0,4077 | 0,3816 | 0,9715 |
| **1-months visit vs 24-months visit** | 0,0236 * | 0,3085 | 0,3373 | 0,7433 | 0,6872 |
| **1-months visit vs 36-months visit** | 0,0022 ** | 0,0499 * | 0,1668 | 0,0788 | 0,6320 |
| **1-months visit vs >36-months visit** | 0,0150 * | 0,1250 | 0,1131 | 0,0349 * | 0,4137 |
| **1-months visit vs Last visit** | 0,0923 | 0,4241 | 0,3441 | 0,5976 | 0,4141 |
| **6-months visit vs 12-months visit** | 0,3882 | 0,3193 | 0,3777 | 0,2420 | 0,9843 |
| **6-months visit vs 24-months visit** | 0,1591 | 0,5396 | 0,3099 | 0,5943 | 0,6812 |
| **6-months visit vs 36-months visit** | 0,0174 * | 0,0937 | 0,1470 | 0,0396 * | 0,6272 |
| **6-months visit vs >36-months visit** | 0,0717 | 0,2091 | 0,1013 | 0,0181 | 0,4061 |
| **6-months visit vs Last visit** | 0,4938 | 0,7401 | 0,3060 | 0,4216 | 0,3889 |
| **12-months visit vs 24-months visit** | 0,5546 | 0,7624 | 0,8506 | 0,5980 | 0,7025 |
| **12-months visit vs 36-months visit** | 0,0932 | 0,3658 | 0,4783 | 0,2846 | 0,6444 |
| **12-months visit vs >36-months visit** | 0,2207 | 0,5474 | 0,3108 | 0,1261 | 0,4196 |
| **12-months visit vs Last visit** | 0,8371 | 0,4965 | 0,9482 | 0,6641 | 0,4163 |
| **24-months visit vs 36-months visit** | 0,2580 | 0,2691 | 0,6047 | 0,1430 | 0,8929 |
| **24-months visit vs >36-months visit** | 0,4352 | 0,4294 | 0,3990 | 0,0630 | 0,6034 |
| **24-months visit vs Last-months visit** | 0,4235 | 0,7500 | 0,8887 | 0,8742 | 0,7190 |
| **36-months visit vs >36-months visit** | 0,8650 | 0,8775 | 0,7091 | 0,5735 | 0,7115 |
| **36-months visit vs Last visit** | 0,0608 | 0,1504 | 0,4899 | 0,1412 | 0,8764 |
| **>36-months visit vs Last visit** | 0,1686 | 0,2933 | 0,3154 | 0,0610 | 0,7690 |

Multiple comparisons were performed by controlling the False Discovery Rate through the two-stage step-up method of Benjamini, Krieger, and Yekutieli. * p<0,05; ** p<0,01; *** p<0,001; **** p<0,0001

**TAB8SM. Impact of previous radiotherapy on disease control during PAS-LAR treatment**

|  | Radiotherapy | No Radiotherapy | P-value |
| --- | --- | --- | --- |
| IGF1-index | | | |
| Baseline | 2,1 ± 1,0 [7] | 1,9 ± 0,6 [43] | 0,493 |
| 1 month | 1,6 ± 1,0 [7] | 1,4 ± 0,7 [26] | 0,557 |
| 6 months | 1,4 ± 0,5 [7] | 1,2 ± 0,5 [42] | 0,371 |
| 12 months | 1,0 ± 0,4 [7] | 1,2 ± 0,5 [36] | 0,414 |
| 24 months | 1,0 ± 0,3 [6] | 1,2 ± 0,6 [27] | 0,398 |
| 36 months | 1,2 ± 0,1 [3] | 0,9 ± 0,3 [16] | 0,124 |
| >36 months | 1,2 [1] | 0,9 ± 0,3 [11] |  |
| Last visit | 0,9 ± 0,3 [7] | 1,2 ± 0,6 [43] | 0,123 |
| Δ IGF1-index between the last and baseline visit | -1,2 ± 1,2 | -0,7 ± 0,6 | 0,327 |
| GH, ng/mL | | | |
| Baseline | 3,0 ± 2,8 [5] | 5,2 ± 6,2 [36] | 0,427 |
| 1 month | 2,6 ± 2,3 [6] | 2,0 ± 1,5 [21] | 0,462 |
| 6 months | 2,9 ± 3,2 [7] | 1,9 ± 1,9 [32] | 0,287 |
| 12 months | 0,9 ± 0,3 [4] | 1,9 ± 2,1 [27] | 0,334 |
| 24 months | 1,2 ± 1,0 [4] | 1,7 ± 2,0 [20] | 0,629 |
| 36 months | 1,4 ± 0,4 [2] | 0,8 ± 0,4 [14] | 0,064 |
| >36 months | - | 0,9 ± 0,4 [11] | - |
| Last visit | 0,9 ± 0,6 [5] | 1,9 ± 2,2 [37] | 0,322 |
| IGF1-index ≤ 1.3, n° (%) | | | |
| Baseline | 2 (29) | 4 (9) | 0,192 |
| 1 month | 2 (29) | 16 (59) | 0,214 |
| 6 months | 4 (57) | 28 (67) | 0,681 |
| 12 months | 5 (71) | 26 (72) | 0,966 |
| 24 months | 6 (100) | 20 (74) | 0,301 |
| 36 months | 3 (100) | 15 (94) | 1,000 |
| >36 months | 1 (100) | 10 (91) | - |
| Last visit | 7 (100) | 28 (65) | 0,087 |
| IGF1-index ≤ 1.0, n° (%) | | | |
| Baseline | 1 (14) | 0 (0) | 0,140 |
| 1 month | 2 (29) | 10 (37) | 0,676 |
| 6 months | 2 (29) | 16 (38) | 0,628 |
| 12 months | 5 (71) | 16 (44) | 0,191 |
| 24 months | 3 (50) | 12 (44) | 0,805 |
| 36 months | 0 (0) | 11 (73) | 0,043 |
| >36 months | 0 (0) | 7 (64) | 0,217 |
| Last visit | 4 (57) | 17 (40) | 0,381 |
| Random GH ≤ 1.0, n° (%) | | | |
| Baseline | 1 (20) | 8 (24) | 0,835 |
| 1 month | 2 (33) | 9 (43) | 0,675 |
| 6 months | 2 (29) | 14 (44) | 0,460 |
| 12 months | 3 (75) | 13 (48) | 0,316 |
| 24 months | 2 (50) | 11 (55) | 0,855 |
| 36 months | 0 (0) | 10 (71) | 0,051 |
| >36 months | - | 7 (70) |  |
| Last visit | 3 (60) | 19 (51) | 0,716 |
| IGF1-index ≤ 1.0 and Random GH ≤ 1.0, n° (%) | | | |
| Baseline | 0 (0) | 0 (0) | - |
| 1 month | 2 (33) | 4 (22) | 0,586 |
| 6 months | 2 (29) | 7 (22) | 0,703 |
| 12 months | 3 (75) | 8 (30) | 0,077 |
| 24 months | 2 (40) | 5 (26) | 0,549 |
| 36 months | 0 (0) | 8 (57) | 0,131 |
| >36 months | - | 6 (60) | - |
| Last visit | 3 (60) | 10 (27) | 0,134 |
| Changes in adenoma dimensions during PAS-LAR therapy, n° (%) | | |  |
| Adenoma reduced | 2 (33) | 14 (45) | 0,069 |
| Adenoma increased | 1 (17) | 0 (0) |  |
| Stable adenoma | 3 (50) | 17 (55) |  |
| Quantitative continuous data are expressed as the mean ±SD. If data do not follow a normal distribution, then the median and interquartile range are reported. Dichotomous and categoric data are shown as numbers and (percentages). For quantitative variables, the sample size is detailed by square brackets. P-values were used to explore the differences observed between subjects who received radiotherapy before starting PAS-LAR and those who did not. Analyses have been conducted by the χ2 test for homogeneity (†) or t-test (‡) as appropriate. Pegvisomant treated patients during follow-up have been excluded from random GH analysis. | | | |

| Tab 9sm. Radiological features and fast recovery from headache during PAS-LAR treatment   \|  \| **Overall [n=50]** \| **PI [n=18]** \| **PD [n=17]** \| **MI [n=15]** \| **p-value** \| \| --- \| --- \| --- \| --- \| --- \| --- \| \| **Pituitary MRI features at baseline** \| \| \| \| \| \| \| Pituitary MRI at baseline, *n° (%)* \| 49 (98) \| 18 (100) \| 16 (94) \| 15 (100) \| 0.371 † \| \| Empty sella or adenoma not visible by MRI, *n° (%)* \| 6 (18) \| 1 (6) \| - \| 5 (33) \| 0.070 † \| \| **Patients with pituitary MRI during follow-up**, *n° (%)* \| 37 (74) \| 17 (94) \| 12 (71) \| 8 (53) \| 0.025 † \| \| **Changes in adenoma dimensions during PAS-LAR therapy** \| \| \| \| \| \| \| Adenoma reduced, *n° (%)* \| 16 (43) \| 9 (53) \| 2 (17) \| 5 (63) \| 0.135 ‡ \| \| Adenoma increased, *n° (%)* \| 1 (3) \| 1 (6) \| 0 (0) \| 0 (0) \| \| Stable adenoma, *n° (%)* \| 20 (54) \| 7 (41) \| 10 (83) \| 3 (37) \| \| **Headache** \| \| \| \| \| \| \| Headache at baseline, n° (%) \| 22 (44) \| 9 (50) \| 9 (47) \| 5 (33) \| 0.600 † \| \| Headache improvement at 1-month, n° (%) ^§^ \| 9 (64) \| 3 (43) \| 3 (75) \| 3 (100) \| 0.195 † \|   Analyses were conducted using the appropriate statistical tests, including Fisher's exact test (†) or the χ2 test for homogeneity (‡). Data regarding the occurrence of empty sella/adenoma not visible by MRI are missing for the Padua cohort. § The percentage of headache improvement was calculated based on subjects who reported having a headache at baseline and completed the 1-month visit (n=14). |
| --- | --- | --- | --- | --- | --- | --- | --- | --- | --- | --- | --- | --- | --- | --- | --- | --- | --- | --- | --- | --- | --- | --- | --- | --- | --- | --- | --- | --- | --- | --- | --- | --- | --- | --- | --- | --- | --- | --- | --- | --- | --- | --- | --- | --- | --- | --- | --- | --- | --- | --- | --- | --- | --- | --- | --- | --- | --- | --- | --- | --- | --- | --- | --- | --- | --- | --- | --- | --- | --- | --- |

**Tab 10SM. Short-term (six months) effects of PAS-LAR dose adjustments (After-Before)**

|  | **Dose decreased by 20 mg /28 d (n=6)** | **Dose increased by 20 mg/28d (n=18)** | **p-value** |
| --- | --- | --- | --- |
| **IGF1-index** | 0.4±0.2 | -0.3±0.4 | 0.002 |
| **Fasting glucose, mg/dL** | -9±6 | 9±12 | 0.004 |
| **HbA1c, mmol/mol** | -4±5 | 2±3 | 0.003 |
| **Glucose Abnormalities** | | | |
| Improvement of glycaemic class / down-titration of DM treatment | 1 (17) | 0 (0) | 0.180 |
| Stable glycaemic class/DM treatment | 4 (67) | 11 (61) |  |
| Worsening of glycaemic class / up-titration of DM treatment | 1 (17) | 7 (39) |  |

During the follow-up period after the introduction of PAS-LAR, 29 dosage modification events were observed, including 10 cases of dosage reduction and 19 cases of dosage increase. Four of these events were excluded from the analysis, comprising three reductions and one increase, as they coincided with the introduction or variation of other acromegaly treatments. Furthermore, one case was excluded due to missing data. The change (delta, Δ) in IGF-1 index, fasting glucose, and HbA1c levels was calculated as the difference between the respective variable's observed values in two visits—one before and the other after the PAS-LAR dosage adjustment. For example, the Δ in the IGF-1 index was calculated by subtracting the IGF-1 index at the pre-adjustment visit from the index at the post-adjustment visit. Consequently, positive Δ values signify an increase in the variable following the PAS-LAR dose adjustment, while negative values denote a decrease. Quantitative data are expressed as mean±SD and analysed by unpaired t-test. Qualitative data are expressed as number (percentage) and evaluated by χ2 test for homogeneity.

**TAB11SM Mixed effect analysis for Assessing Quantitative Parameters of Acromegaly's Biochemical Control and Glucose Metabolism Status. Multiple Comparisons**

|  | **IGF-index**  **p-value** | **Random GH**  **p-value** | **Fasting Glucose**  **p-value** | **HbA1c**  **p-value** |
| --- | --- | --- | --- | --- |
| **Baseline vs 1-month visit** | 0,0005 *** | 0,3449 | 0,0006 *** | 0,0003 *** |
| **Baseline vs 6-months visit** | <0,0001 **** | 0,1369 | <0,0001 **** | <0,0001 **** |
| **Baseline vs 12-months visit** | <0,0001 **** | 0,2439 | <0,0001 **** | <0,0001 **** |
| **Baseline vs 24-months visit** | <0,0001 **** | 0,0012 ** | 0,0337 * | 0,0133 * |
| **Baseline vs 36-months visit** | <0,0001 **** | 0,0038 ** | 0,0191 * | 0,0066 ** |
| **Baseline vs >36-months visit** | 0,0006 *** | 0,0940 | 0,0535 | 0,0236 * |
| **Baseline vs Last visit** | <0,0001 *** | 0,0447 * | <0,0001 **** | <0,0001 **** |
| **1-months visit vs 6-months visit** | 0,4794 | >0,9999 | 0,8932 | >0,9999 |
| **1-months visit vs 12-months visit** | 0,2610 | 0,9739 | >0,9999 | >0,9999 |
| **1-months visit vs 24-months visit** | 0,1813 | 0,8959 | 0,9621 | 0,9975 |
| **1-months visit vs 36-months visit** | 0,0620 | 0,0491 * | 0,9987 | 0,9944 |
| **1-months visit vs >36-months visit** | 0,2006 | 0,2779 | 0,9671 | 0,8808 |
| **1-months visit vs Last visit** | 0,5116 | 0,8928 | 0,9809 | 0,8019 |
| **6-months visit vs 12-months visit** | 0,6764 | 0,7689 | 0,8711 | 0,9953 |
| **6-months visit vs 24-months visit** | 0,9641 | 0,8838 | 0,2682 | 0,9929 |
| **6-months visit vs 36-months visit** | 0,0485 * | 0,0133 * | >0,9999 | 0,9669 |
| **6-months visit vs >36-months visit** | 0,2986 | 0,1856 | >0,9999 | 0,6723 |
| **6-months visit vs Last visit** | 0,9997 | 0,8505 | >0,9999 | 0,3888 |
| **12-months visit vs 24-months visit** | >0,9999 | 0,9928 | 0,7274 | 0,7306 |
| **12-months visit vs 36-months visit** | 0,0904 | 0,0214 * | 0,9961 | 0,9863 |
| **12-months visit vs >36-months visit** | 0,3956 | 0,0612 | 0,8968 | 0,5588 |
| **12-months visit vs Last visit** | 0,8849 | >0,9999 | 0,9200 | 0,5132 |
| **24-months visit vs 36-months visit** | 0,0774 | 0,0290 * | 0,2990 | 0,0832 |
| **24-months visit vs >36-months visit** | 0,0433 * | 0,2045 | 0,1218 | 0,1244 |
| **24-months visit vs Last-months visit** | 0,3176 | 0,7934 | 0,0290 * | 0,0010 ** |
| **36-months visit vs >36-months visit** | >0,9999 | >0,9999 | 0,9953 | 0,9305 |
| **36-months visit vs Last visit** | 0,0101 * | 0,0075 ** | >0,9999 | 0,9961 |
| **>36-months visit vs Last visit** | 0,0175 * | 0,0483 * | 0,9965 | 0,9850 |

Multiple comparisons were performed by Tukey’s multiple comparisons test. * p<0,05; ** p<0,01; *** p<0,001; **** p<0,0001

**TAB12SM Multiple comparison test for quantitative continuous variables of Table 3**

|  | **ANOVA summary** | |  |  |  |  |  |  |
| --- | --- | --- | --- | --- | --- | --- | --- | --- |
|  | **F-value (df)** | **p-value** | **PI vs PD** | | **PI vs MI** | | **PD vs MI** | |
|  |  |  | **Mean Diff. (95% CI)** | **Adj. p-value** | **Mean Diff, (95% CI)** | **Adj. p-value** | **Mean Diff, (95% CI)** | **Adj. p-value** |
| **IGF-1 index** | | | | | | | | |
| Baseline | 6,464 (2,47) | 0,003 | 0,5 (0,0 to 0,9) | 0,063 | -0,3 (-0.8 to 0,3) | 0,382 | -0,7 (-1,2 to -0,2) | 0,003 |
| 1 month | 0,120 (2,31) | 0,887 |  |  |  |  |  |  |
| 6 months | 0,154 (2,46) | 0,856 |  |  |  |  |  |  |
| 12 months | 0,251 (2,40) | 0,779 |  |  |  |  |  |  |
| 24 months | 1,363 (2,30) | 0,271 |  |  |  |  |  |  |
| 36 months | 0,482 (2,16) | 0,626 |  |  |  |  |  |  |
| >36 months | 5,278 (2,9) | 0,026 | -0,5 (-1,0 to -0,1) | 0,026 | 0,0 (-0,7 to 0,7) | 1,000 | 0,5 (-0,3 to 1,3) | 0,207 |
| Last visit of the patient | 0,295 (2,47) | 0,74 |  |  |  |  |  |  |
| **Random GH** | | | | | | | | |
| Baseline | 1,691 (2,35) | 0,199 |  |  |  |  |  |  |
| 1 month | 0,372 (2,24) | 0,693 |  |  |  |  |  |  |
| 6 months | 0,041 (2.36) | 0,960 |  |  |  |  |  |  |
| 12 months | 0,183 (2,28) | 0,834 |  |  |  |  |  |  |
| 24 months | 0,050 (2,21) | 0,952 |  |  |  |  |  |  |
| 36 months | 1,244 (2,13) | 0,320 |  |  |  |  |  |  |
| >36 months |  | - |  |  |  |  |  |  |
| Last visit of the patient | 0,195 (2,39) | 0,824 |  |  |  |  |  |  |
| **Δ IGF1-index between Last and baseline visit** | 5,901 (2,47) | 0,005 | -0,6 (-1,2 to 0,0) | 0,037 | -0,3 (-0,4 to 0,8) | 0,683 | 0,7 (0,2 to 1,4) | 0,006 |
| **Δ IGF1-index between Last and 1-month visit** | 0,658 (2,31) | 0,525 |  |  |  |  |  |  |
| **Δ IGF1-index between the last and first* visit** | 1,984 (2,47) | 0,149 |  |  |  |  |  |  |
| **Δ IGF1-index between the 1-month and baseline visit** | 3,421 (2,31) | 0,046 | -0,4 (-1,1 to 0,3) | 0,290 | -0,3 (-0,9 to 0,9) | 0,403 | 0,7 (0,0 to 1,4) | 0,036 |
| **Δ IGF1-index between the first* and baseline visit** | 5,564 (2,47) | 0,007 | -0,2 (-0,6 to 0,3) | 0,679 | 0,5 (0,0 to 1,0) | 0,046 | 0,7 (0,2 to 1,1) | 0,006 |

Df (between groups, within groups)

**TAB13SM Multiple comparison test for quantitative continuous variables of Table 5**

|  | **ANOVA summary** | |
| --- | --- | --- |
|  | **F-value (df)** | **p-value** |
| **Fasting Glucose** | | |
| Baseline | 0,554 (2,47) | 0,579 |
| 1 month | 0,645 (2,30) | 0,532 |
| 6 months | 0,438 (2,40) | 0,648 |
| 12 months | 0,187 (2,36) | 0,831 |
| 24 months | 0,096 (2,27) | 0,909 |
| 36 months | 1,259 (2,15) | 0,312 |
| > 36 months | 1,045 (2,9) | 0,391 |
| Last visit of the patient | 0,867 (2,41) | 0,428 |
| **HbA1c** | | |
| Baseline | 0,534 (2,47) | 0,590 |
| 1 month | 0,564 (2,31) | 0,564 |
| 6 months | 0,243 (2,44) | 0,785 |
| 12 months | 0,537 (2,40) | 0,588 |
| 24 months | 0,286 (2,28) | 0,754 |
| 36 months | 1,228 (2,16) | 0,319 |
| > 36 months | 0,301 (2,9) | 0,747 |
| Last visit of the patient | 0,033 (2,45) | 0,967 |

Df (between groups, within groups)

**Figure 1SM. PAS-LAR dose titration**


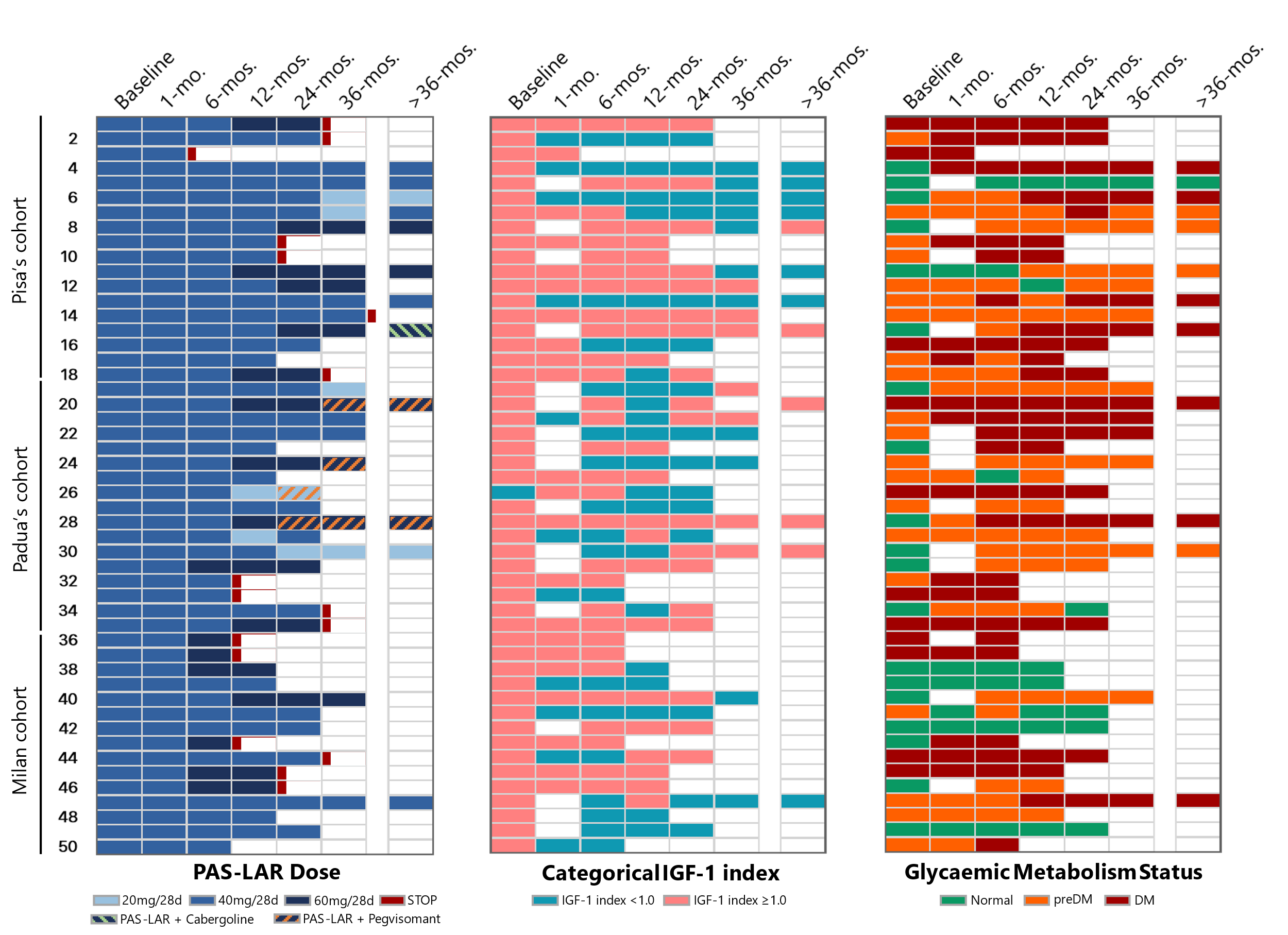


**Figure 2SM. IGF-1 index values for each patients during follow-up**

**
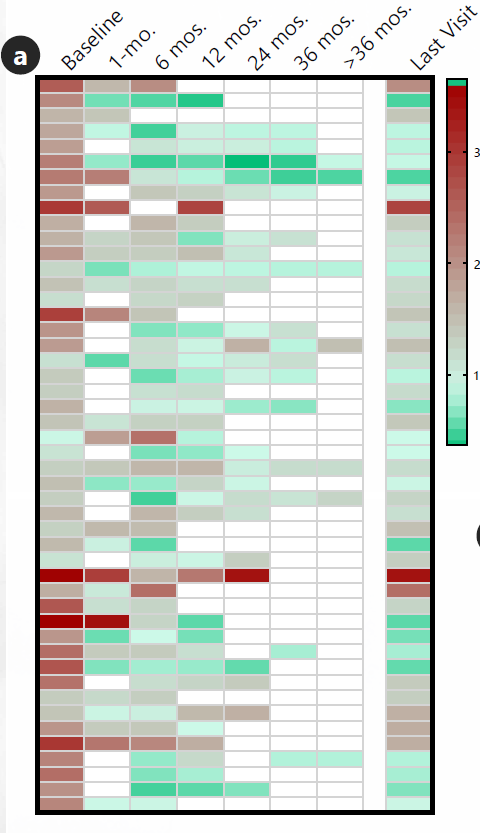
**

**Fig 3SM. Symptoms control after PASLAR start**

**
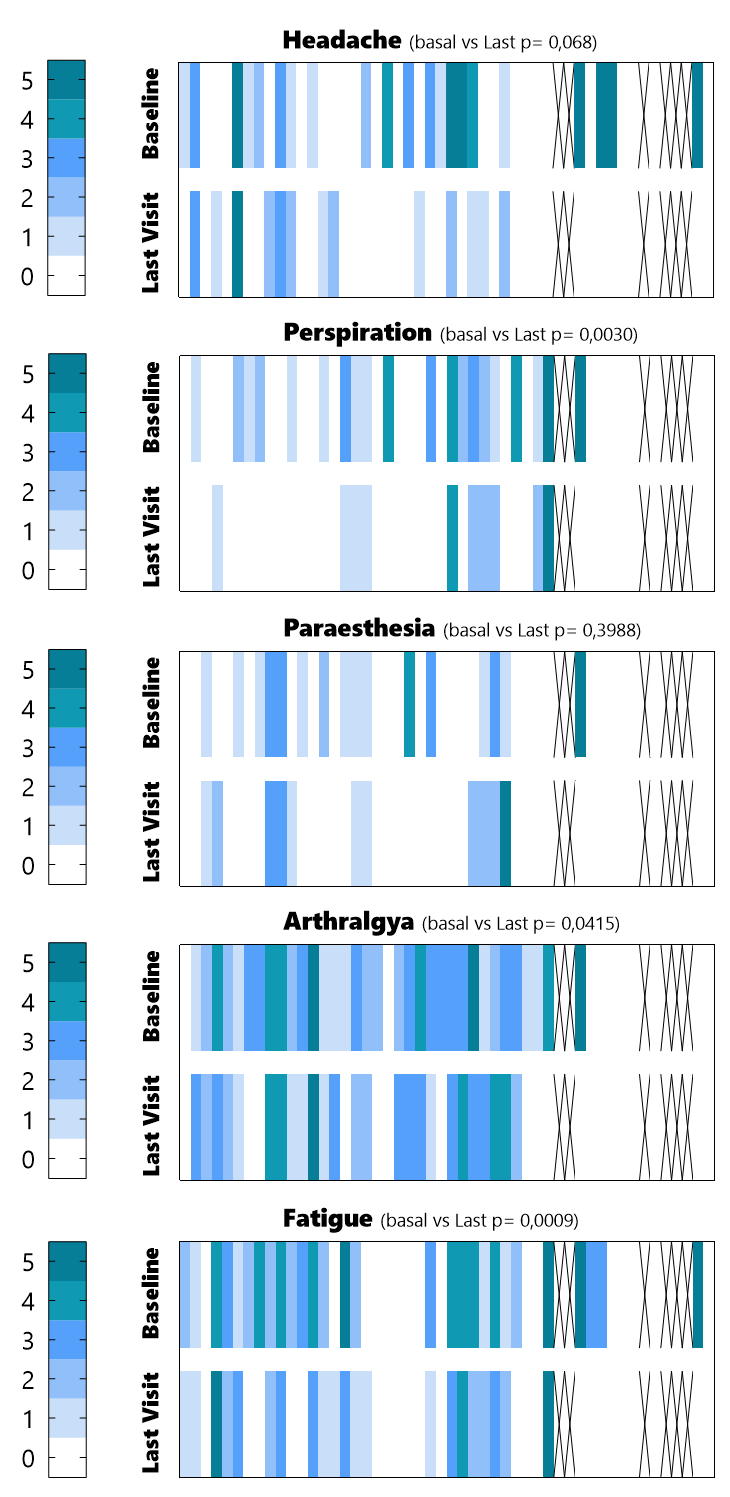
**

**Figure 4SM. Patients divided into different metabolism classes.**


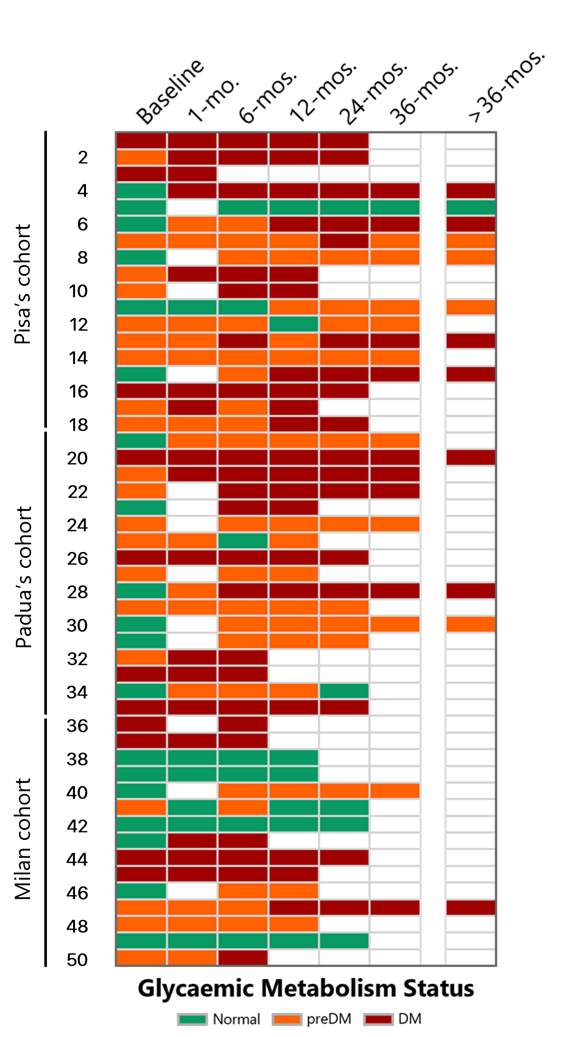

Supplement: Supplementary file 1 — Supplementary file1 (DOCX 885 KB) [file 40618_2023_2275_MOESM1_ESM.docx]
